# Crime and Psycho-Analysis

**Published:** 1921-04-02

**Authors:** 


### CRIME AND PSYCHO-ANALYSIS.

It was almost inevitable that sooner or later the extravagant theories put forward by the more advanced or cocksure exponents of psycho-analysis would receive a rebuff when they sought to introduce into courts of law their fantastic arguments for relieving the criminal of the responsibility for his crime. We think that the common-sense which still distinguishes human beings in the mass will approve the contemptuous way in which a few days ago the Court of Appeal dismissed an appeal (based on the new science) against a conviction for murder. The defence, already becoming common in this country when the evidence is irresistible, was that the crime was committed in a moment of insanity as the result of an irresistible impulse, and mental experts were called in the lower court to describe the state in which they had found the criminal's sub-conscious mind, and to explain how it was, through forces beyond his own control, that he had been driven to commit a particularly brutal murder. There can be nothing more irritating to a robust, decent-minded man than to be expected to give serious attention to the excuses, based on some dim hypothesis about an obscure science, that a crime, which was clearly the outcome of careful premeditation, was successfully accomplished while the perpetrator was "in a dream," when he was "quite obsessed," when "something seemed to possess him," and so forth, *ad nauseam*.

At the trial one of the doctors said he sought to find out whether, when the accused man had the knife with which he committed the murder sharpened on both sides, it "symbolised" any idea in his mind. For the benefit of the Appeal Court it was explained that a sane man who contemplated murder would not sharpen both edges because one would be sufficient! It had evidently not occurred to this sapient expert until somebody pointed it out to him that as the swords of cavalry regiments are sharpened on both sides the military authorities who issue the order must also be dangerously insane. In this case everything possible seems to have been done to try to prove that any murderer is *ipso facto* insane. The prison authorities even went so far as to allow a doctor called for the defence to hypnotise the prisoner, and then question him about the crime—a precedent open to grave objection, for, clearly, if a man in such circumstances may be examined for evidence under one kind of abnormal condition he may be examined under another kind—for instance, while he is under the influence of drugs—and, if the defence may adopt such a course, why not the prosecution? In refusing to attach any importance to the medical evidence for the defence, Mr. Justice Darling, who presided in the Court of Appeal, declared, without unnecessary verbiage, that "the law of England had not recognised the theory of all these slabs of intelligence, beginning with the unconscious base and finishing with the conscious mind"; and the Court upheld Mr. Justice Acton's ruling that the medical evidence was based on "speculative theories which had not yet met general acceptance."

This judgment is, of course, open to the retort, which has been quickly forthcoming, that legal opinion does not affect scientific fact; but what murderer would ever be hanged or punished at all if the appeal had been allowed upon the theory of the psycho-analysts? and how far, it may be asked, is a man to be permitted to plead the involuntary operation of his sub-conscious mind when he is discovered in a wicked, illegal, or immoral act for which by the ordinary laws of man and according to the universal sanction of morality he would be held responsible? Are we to say that the man whose will is mastered at a given moment by an evil passion is invariably mad? Even assuming the correctness of the scientific theory, what the proposition seems to amount to is this: that evidence of premeditation, instead of being regarded as deepening the guilt, would be accepted, as one writer puts it, as a mental process on the way to the irresistible impulse of insanity. That would merely be reducing the law to a grotesque absurdity, for upon that reasoning every murderer who has met his death by a verdict of his fellow-countrymen has been unjustly hanged. Whatever benefits psycho-analysis may ultimately bestow upon the science of medicine, or whatever new light it may throw on mental disease, we do not think it will allow intelligent men to be persuaded that crime ought to be called by another name, or that it will ever cause the punishment of crime to be expunged from the judicial code.

